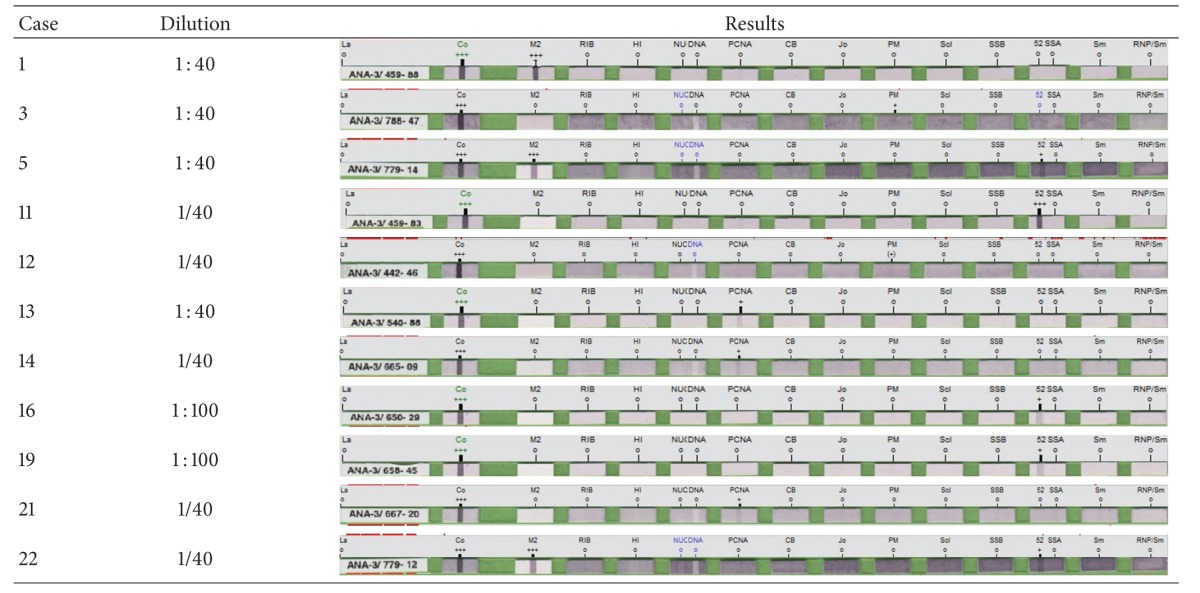# Idiopathic Granulomatous Mastitis: An Autoimmune Disease?

**DOI:** 10.1155/2013/148727

**Published:** 2013-09-04

**Authors:** Fatih Altintoprak, Engin Karakece, Taner Kivilcim, Enis Dikicier, Guner Cakmak, Fehmi Celebi, Ihsan Hakkı Ciftci

**Affiliations:** ^1^Department of General Surgery, Sakarya University Faculty of Medicine, Sakarya, Turkey; ^2^Department of Microbiology, Sakarya University Faculty of Medicine, Sakarya, Turkey; ^3^Department of General Surgery, Sakarya University Research and Educational Hospital, Sakarya, Turkey

## Abstract

*Purpose*. This study aimed to investigate the autoimmune basis of idiopathic granulomatous mastitis (IGM) by determining the anti-nuclear antibody (ANA) and extractable nuclear antigen (ENA) levels of patients diagnosed with IGM. *Material and Methods*. Twenty-six IGM patients were evaluated. Serum samples were analyzed for autoantibodies by indirect immunofluorescence (IIF) using a substrate kit that induced fluorescein-conjugated goat antibodies to human immunoglobulin G (IgG). IIF patterns were read at serum dilutions of 1 : 40 and 1 : 100 for ANA positivity. Using the immunoblot technique, the sera of patients were assayed at dilutions of 1 : 40 and 1 : 100 for human autoantibodies of the IgG class to 15 lines of highly purified ENAs. *Results*. In the IIF studies for ANA, positivity was identified for four different patterns in the 1 : 40 diluted preparations, for three different patients in the 1 : 100 diluted preparations and only one pattern was identified at the 1 : 320 dilution. In the ENA studies, positivity was identified for four different pattern in the 1 : 40 dilution, and only one pattern was identified at the 1 : 100 dilution. *Conclusion*. This study was not able to support the eventual existence of an autoimmune basis for IGM.

## 1. Introduction

 Idiopathic granulomatous mastitis (IGM) is a rarely observed, chronic inflammatory breast disease of unknown etiology that can clinically and radiologically mimic breast cancer [1]. Observation of noncaseous granulomas during histopathological evaluation is characteristic of the disease and is considered the diagnosis criteria for IGM after other potential infectious causes (such as tuberculosis and certain mycoses) and noninfectious causes (such as sarcoidosis and vasculitis) have been excluded [2].

 This benign disease may present itself with various clinical findings associated with breast tissue (e.g., a palpable mass, nipple retraction, inflammation-erosion on the breast skin and fistulae) [3]. Although various methods have been used for IGM treatment (such as surgical excision, steroids, methotrexate, and close followup), no consensus currently exists regarding the ideal method of treatment. 

 Despite various explanations that have been proposed, the exact etiological factors of IGM have not yet been elucidated. Due to its positive response to steroid treatment, the hypothesis that IGM is an autoimmune disease is currently the most commonly accepted view. In the present study, the stages of diagnosis, clinical findings, and treatment outcomes of 26 patients diagnosed with IGM are presented, and the autoimmune basis of the disease is discussed by assessing antinuclear antibody (ANA) and extractable nuclear antibody (ENA) levels. 

## 2. Material and Methods

The records of 26 female patients diagnosed with IGM and tested for ANA-ENA levels between January 2007 and January 2013 at the Sakarya University Medical Faculty General Surgery Clinic were analyzed retrospectively. The patients' demographic characteristics, stages of diagnosis, administered treatments, and ANA-ENA results were evaluated in detail within the records. 

### 2.1. Diagnostic Procedures

Regarding patient history, a detailed review was performed of the complaints at admission, the duration of the complaints, whether the complaints were of a repetitive nature, the treatments used for these complaints, the number of pregnancies, the duration of nursing, smoking habits, oral contraceptive use, and the presence of chronic systemic autoimmune disease. Thereafter, the physical examination findings of the patients (such as the presence of a mass in the breast, inflammation findings, and fistulae) were recorded.

### 2.2. Imaging Methods

Breast ultrasonography (USG) was performed for all patients, whereas mammography and magnetic resonance imaging (MRI) were used depending on the age and clinical condition of the patients. Mammographic evaluation was performed in accordance with the Breast Imaging Reporting and Data System (BIRADS) criteria.

### 2.3. Tissue Sampling Procedures

Fine-needle aspiration biopsy (FNAB), Tru-cut biopsy, or incisional or excisional biopsy was performed depending on the clinical findings at the time of admission. For all biopsy specimens, Gram, periodic acid-Schiff (PAS), and Ziehl-Nielsen staining procedures were performed for the analysis of microbiological agents, and culture methods were used for tuberculosis and fungi. 

### 2.4. Differential Diagnosis Procedures

Thoracic imaging studies (posterior-anterior chest X-ray or computed tomography) and purified protein derivative (PPD) skin tests were performed in all patients. A diagnosis of granulomatous mastitis was established by histopathological examination showing the presence of numerous epithelioid cells as well as the multinucleated Langerhans-type giant cells, neutrophils, lymphocytes, and stromal cells in the FNAB samples and the presence of an exclusively granulomatous inflammatory reaction with neither caseous necrosis nor any specific organism in the samples obtained by other biopsy methods.

### 2.5. Measurement of ANA and ENA Levels

Serum samples were analyzed for autoantibodies by indirect immunofluorescence (IIF) using a substrate kit (EUROIMMUN, Germany) that induced fluorescein-conjugated goat antibodies to human immunoglobulin G (IgG). IIF patterns were read at serum dilutions of 1 : 40 and 1 : 100 for ANA positivity on a Zeiss Axioskop microscope (Carl Zeiss, Jena, Germany) by the same experienced microbiologist. Titers of 1 : 40 were used as a primary screening. Titers of at least 1 : 100 were regarded as positive, and different nuclear and cytoplasmic fluorescence patterns were documented. Sera of patients with IGM were assayed at dilutions of 1 : 40 and 1 : 100 for human autoantibodies of the IgG class to 15 lines of highly purified ENAs (M2, Rib, HI, NU, DNA, PCNA, CB, Jo, PM, Scl, SSB, Ro-52, SSA, Sm, and RNP/Sm) using an immunoblot technique and the manufacturer's instructions (ANA-Profile 3, EUROIMMUN, Germany). ENA results were obtained using EUROlineScan software (EUROIMMUN, Germany). 

### 2.6. Treatment Procedures

Patients with inflammatory findings, but without abscess findings, were initially treated with an antibiotic and an anti-inflammatory drug for an average of 14 days (range: 10–21 days). USG-assisted aspiration or surgical drainage was performed in patients with abscess findings at the time of diagnosis and in whom the diagnosis of abscess was confirmed by USG examination. Surgical excision was performed in patients with an isolated mass without the presence of skin changes. Steroid treatment was mainly preferred in patients in whom the breast skin was also affected by extensive inflammation, fistulae, or erosion, and/or extensive parenchymal involvement was confirmed by MRI examination.

## 3. Results

The mean age of the patients was determined to be 37.5 years (range: 24–65 years). All patients had given live births at least once (range: 1–8) and had nursed for an average of 9.5 months (range: 3–21 months). Six patients (23.0%) had a history of smoking, whereas three patients (11.5%) had a history of oral contraceptive use. At the time of diagnosis, none of the patients were pregnant, lactating, or had a history of breast trauma, galactorrhea, chronic systemic autoimmune disease, or regular steroid use. It was determined for all patients that the initial complaints began following the birth and nursing periods and that there were no complaints in the prepregnancy period. Interestingly, four of the patients (15.3%) had a history of not nursing from the breast in which the IGM later developed (due to infants' unwillingness to nurse from this breast, although they nursed normally from the other breast). The mean duration between the end of lactation and the initial complaint was 3.2 years (range: 2–6 years) for these four patients. 

 Unilateral involvement was present in all patients (the right breast in 14 (53.8%) patients and the left breast in 12 (46.2%) patients). The most frequent complaints at admission (16 (61.5%) patients) were pain, swelling, and inflammation on the affected breast, along with superficial erosion or open fistulae on the breast skin. In 10 (38.4%) patients, a palpable mass in the breast was the initial complaint at admission. The complaints of patients admitted for inflammatory changes in the breast were of a repetitive nature, and these patients had histories of intermittent antibiotic and anti-inflammatory drug use due to these complaints for an average period of 7 months (3–16 months). 

 Surgical treatment was administered in 10 patients (38.4%), with wide excisions performed in eight of the patients (8/10, 80%) and quadrantectomy performed in two of the patients (2/10, 20%). In two of the patients (2/10, 20%) who received surgical treatment, relapse occurred at an average of 29.1 months (range: 22–53 months) into followup. These patients received steroid treatment. In two of the patients administered steroids (2/16, 12.5%), relapse occurred at an average of 15.7 months (range: 6–47 months) into followup. These patients were also treated with steroids. 

 During the indirect fluorescent microscopy studies for ANA, four different patterns were identified in the 1 : 40 diluted preparations; nine (34.6%) patients had nucleolar positivity, nine (34.6%) patients had nuclear speckled positivity, two (7.6%) patients had cytoplasmic positivity, and two (7.6%) patients had mitotic spindle positivity. Negativity was identified for five (19.2%) of the patients during the evaluations performed at the 1 : 40 dilution. At the 1 : 100 dilution, which is frequently used in screening tests for autoimmune diseases, three different patterns were identified; four (15.3%) patients had nucleolar positivity, three (11.5%) patients had nuclear speckled positivity, and two (7.6%) patients had mitotic spindle positivity. Negativity was identified for 17 (65.3%) patients during the evaluations performed at the 1 : 100 dilution. Only one (3.8%) patient was identified with both nucleolar and nuclear speckled positivity (Case 5), whereas two (7.6%) patients were identified with nucleolar positivity at the 1 : 320 dilution.

 In the ENA studies performed at the 1 : 40 dilution using the immunoblot technique, five (19.2%) patients were identified with Ro-52 positivity, three (11.5%) patients were identified with PCNA positivity, two (7.6%) patients were identified with PM positivity, and one (3.8%) patient was identified with M2 positivity. However, negative results were identified for 15 (57.6%) patients. In the ENA tests performed at the 1 : 100 dilution to confirm the ANA screening tests and for detailed antibody positivities, only two (7.6%) patients were identified with Ro-52 positivity, whereas negative results were identified in 24 (92.3%) patients. 

 The distribution of ANA and ENA patterns are shown in Table 1. 

 Final results for ENA positivity in patients with IGM are shown in Table 2.

## 4. Discussion

IGM is a rarely observed chronic disease that can clinically mimic breast cancer or abscesses and relapse after treatment. The clinical course of IGM is troublesome for both clinicians and patients, particularly in cases when the disease recurs. Although IGM is a benign disease, first identified more than 40 years ago, an ideal method of treatment has still not been described. This arises because the etiology of the disease has not yet been fully elucidated. The different rates of recurrence reported for each treatment method have been previously described for IGM [4, 5], and one of the treatment approaches only involves close regular clinical surveillance [6], supporting our view concerning the lack of an ideal treatment method. 

 As IGM is a disease that involves the breast in an isolated manner, its mechanism of development is believed to involve the following sequence: ductal epithelial damage, transition of luminal secretions to the lobular connective tissue, local inflammation in connective tissue, macrophage and lymphocyte migration to the region, and local granulomatosis inflammatory response [7]. However, the trigger factor in the development of epithelial damage has not been clarified. Autoimmunity, pregnancy, lactation, hyperprolactinemia, oral contraceptive use, local trauma to the breast, alpha-1 antitrypsin deficiency, rarely observed infectious factors, local irritants, smoking, and diabetes mellitus are believed to be trigger factors in IGM etiology [8–10]. 

 Although the association of cigarette and oral contraceptive use with IGM is mentioned in numerous studies, their involvement as risk factors has not been conclusively demonstrated [11, 12]. Hyperprolactinemia has been described to potentially play a role in the development of IGM by causing overstimulation of the breast parenchyma as well as changes similar to the lactation period [13]. Erhan et al. [4] reported recurrence in three (16%) patients from their 18-patient series and identified hyperprolactinaemia in two of these patients. Bani-Hani et al. [10], conversely, reported that they measured serum prolactin levels in seven of the patients from their 24-patient series and identified high serum prolactin values in only one of the patients. In our study, the ratios of smoking and oral contraceptive use were determined to be 23% and 11.5%, respectively, whereas none of the patients had a history of galactorrhea. 

 Presently, particular emphasis is being placed on IGM being of autoimmune origin. In the literature, the favorable response to steroid and immunosuppressive treatments, the favorable response to steroid treatment observed in patients with postsurgical treatment recurrence, the description of patients with extramammary involvement such as erythema nodosum or arthritis, and the demonstration of T-lymphocyte predominance in immunohistochemical studies are described as findings that support the autoimmune hypothesis [4, 5, 10, 14–17]. However, classical serological tests used in autoimmune disorders (such as ANA and rheumatoid factor (RF)) provide different results in IGM patients. Asoglu et al. [18] demonstrated negative ANA and RF levels in their 18-patient series, whereas Ozel et al. [19] reported RF and ANA positivity for six and two patients, respectively, in their eight-patient series.

 Only ANA positivity was not specific for autoimmune disease because positivity may occur in patients with other diseases or even in healthy subjects. The prevalence of low-titer ANA positivity is variable in healthy populations. Furthermore, elderly female individuals over 60 years of age have relatively high frequencies of ANA. It is estimated that 10–15% of healthy people over 65 years of age have ANA positivity, and titers are usually higher than 1 : 100. In the present study, it was accepted that 1 : 40 was the low titer for ANA and ENA positivity, whereas 1 : 100 was the lowest titer for ANA and ENA positivity [20]. Among the 26 patients with IGM, 21 (80.8%) had ANA positivity at 1 : 40 titers, 9 (34.6%) had ANA positivity at 1 : 100 titers, and 2 (7.8%) had moderate ANA positivity at 1 : 320 titers. A possible explanation for the results presented in the present study is that the positivity rate could be the normal population value. Thus, none of the patients were found to have titers with higher ANA positivity. Similarly, 11 (42.3%) patients had ENA positivity at 1 : 40 titers, whereas 2 (7.8%) had low ENA positivity at 1 : 100 titers. This result is not surprising because most of the patients had low ANA titers. It is interesting to note that both the lowest and low ENA titers were demonstrated to have Ro-52 positivity. Ro-52 has been previously shown to be the prevalent autoantibody in systemic autoimmune diseases such as systemic sclerosis and systemic lupus erythematosus. In addition, Ro-52 plays an important role in antiviral defense, cell cycle regulation, cellular activation, and the regulation of inflammatory cytokine production. Therefore, in addition to being a target for autoantibody production, Ro-52 also assumes a critical role in immune regulation [21, 22]. 

 Despite the reporting of several nulliparous or nonreproductive cases [6, 10], a review of the literature reveals that IGM patients are generally reproductive female patients between 30 and 45 years of age with a history of pregnancy in the past 5 years (range: from 2 months to 20 years). All of the cases in our study (except one aged 65) were in their reproductive period, had given live births at least once, had nursed for certain periods of time, and had no complaints prior to their first delivery. Moreover, due to complaints pertaining to the inability to nurse from the breast that would later be diagnosed with IGM (an interesting observation that we have noted), we believe that pregnancy and lactation both occupy a significant place among the potential trigger factors for the development of IGM. 

In conclusion, the present study assessed the presence of definite autoantibodies associated with autoimmunity in patients with IGM. However, a predominant ANA and ENA indicator that demonstrated the role of autoimmune factors in the etiology of IGM could not be identified. However, long-term followup of Ro-52-positive patients at both 1 : 40 and 1 : 100 dilutions can provide further information concerning the autoimmune basis of IGM. 

## Figures and Tables

**Table 1:** Distribution of ANA patterns and ENA positivity in patients with IGM.

Patient no.	Anti-nuclear antibody		Anti-extractable nuclear antibody	
	1/40	1/100	1/40	1/100
1	Cytoplasmic	—	M2	—
2	Nucleolar	Nucleolar (1/320)	—	—
3	Cytoplasmic	—	PM	—
4	—	—	—	—
5	Nucleolar and nuclear speckled	Nucleolar (1/320)	Ro-52	—
6	Nucleolar	Nucleolar	—	—
7	Nucleolar	Nucleolar	—	—
8	Nucleolar	—	—	—
9	Nucleolar	—	—	—
10	Nucleolar	—	—	—
11	Nuclear speckled	—	Ro-52	—
12	Nucleolar	—	PM	—
13	Nucleolar	—	PCNA	—
14	Nuclear speckled	—	PCNA	—
15	—	—	—	—
16	Nuclear speckled	Nuclear speckled	Ro-52	Ro-52
17	Mitotic spindle	Mitotic spindle	—	—
18	—	—	—	—
19	Nuclear speckled	Nuclear speckled	Ro-52	Ro-52
20	Mitotic spindle	Mitotic spindle	—	—
21	Nuclear speckled	Nuclear speckled	PCNA	—
22	Nuclear speckled	—	Ro-52	—
23	—	—	—	—
24	—	—	—	—
25	Nuclear speckled	—	—	—
26	Nuclear speckled	—	—	—

**Table 2:** Final results for ENA positivity in patients with IGM.